# Standardizing to specific target populations in distributed networks and multisite pharmacoepidemiologic studies

**DOI:** 10.1093/aje/kwae015

**Published:** 2024-02-27

**Authors:** Michael Webster-Clark, Kristian B Filion, Robert W Platt

**Keywords:** distributed networks, standardization, external validity, target populations

## Abstract

Distributed network studies and multisite studies assess drug safety and effectiveness in diverse populations by pooling information. Targeting groups of clinical or policy interest (including specific sites or site combinations) and applying weights based on effect measure modifiers (EMMs) prior to pooling estimates within multisite studies may increase interpretability and improve precision. We simulated a 4-site study, standardized each site using inverse odds weights (IOWs) to resemble the 3 smallest sites or the smallest site, estimated IOW-weighted risk differences (RDs), and combined estimates with inverse variance weights (IVWs). We also created an artificial distributed network in the Clinical Practice Research Datalink (CPRD) Aurum consisting of 1 site for each geographic region. We compared metformin and sulfonylurea initiators with respect to mortality, targeting the smallest region. In the simulation, IOWs reduced differences between estimates and increased precision when targeting the 3 smallest sites or the smallest site. In the CPRD Aurum study, the IOW + IVW estimate was also more precise (smallest region: RD = 5.41% [95% CI, 1.03-9.79]; IOW + IVW estimate: RD = 3.25% [95% CI, 3.07-3.43]). When performing pharmacoepidemiologic research in distributed networks or multisite studies in the presence of EMMs, designation of target populations has the potential to improve estimate precision and interpretability.

**This article is part of a Special Collection on Pharmacoepidemiology**.

## Introduction

Distributed networks and multisite studies are important resources for pharmacoepidemiologic studies of drug safety and effectiveness.^1–4^ In a typical distributed network, a coordinating center maintains relationships with multiple data partners (ie, “nodes” or sites) that retain ownership and custody of individual-level patient data and conduct analyses independently. Current distributed networks include the Sentinel System (created by Harvard Pilgrim Health Care and the Food and Drug Administration),^1^^,^^5^ the Canadian Network for Observational Drug Effect Studies (CNODES),^6^ the National Patient-Centered Clinical Research Network (PCORnet),^7^ the Data Analysis and Real World Interrogation Network (DARWIN EU),^8^ and the network maintained by the Observational Health Data Sciences and Informatics (OHDSI) group.^9^ Each studies drug safety and effectiveness in diverse real-world populations by conducting studies at multiple sites and pooling findings. One-off multisite studies similarly have investigators at individual study sites generate estimates for subsequent combination.

Diversity of patient characteristics and underlying populations can make synthesizing evidence difficult, however.^3^ Whether directly combining analytical data or meta-analyzing estimates, researchers must decide how to handle outlier estimates when consolidating results. While these decisions are challenging even if treatment effects are uniform, they become even more complex in the presence of treatment effect heterogeneity and effect measure modification.^10^^,^^11^ Moreover, if treatment effects within specific sites are of substantive interest (say, if each site represents a unique province, country, or insurance provider where drug safety and effectiveness is of interest), the precise consolidated estimate may not reflect every target population.^12^^,^^13^ Using estimates within sites without any combination or meta-analysis, however, can lead to imprecise and uninformative estimates for smaller sites.

When sites differ in the prevalence or distribution of effect measure modifiers (EMMs), ignoring them can also mask the similarity of results across nodes. Suppose a side effect only occurs in adults over 65 years of age. While 30% of one node of the network is over age 65, 60% of another node is over age 65. Even if analyses in both nodes removed all confounding and agreed about the effect in persons under and over 65 years of age, population-level estimates would still differ; indeed, if there is age-based modification of the drug’s effects, we should be *concerned* if the population-level estimates are identical in these 2 populations. This is likely when either some sites comprise almost entirely older adults (eg, the US Medicare program) or some data are only available for older adults (eg, prescription drug data in Ontario, Canada). In this particular case, we would be better served by comparing and meta-analyzing subgroup estimates.

When faced with large numbers of EMMs, continuous EMMs, or small sites, however, subgroup-based approaches become intractable.^14^^,^^15^ There is an alternative: weighting each site to a common target population with a known distribution of covariates and EMMs. Because every site postweighting will have similar distributions of measured EMMs (provided correct weights), differences between sites’ weighted estimates result from unmeasured confounders, unmeasured EMMs, or both. Moreover, because each analysis is estimating treatment effects in the same population, random-effects meta-analysis is less important.^16^ Further, varying the target population across the sites of a multisite study or distributed network can help one examine effects at sites relevant to the stakeholders participating in the network, akin to analyzing a variety of target populations for randomized controlled trials.^17^

These analytical methods are not unknown in epidemiology and pharmacoepidemiology and are closely related to ongoing developments in “causal meta-analysis” of randomized trials.^18^^,^^19^ That type of work aims to clarify and better operationalize estimands of meta-analyses by creating methods combining treatment effects from multiple studies from different populations that vary in “case mix” (ie, distributions of covariates) into an estimate in a defined target population,^20^ as well as estimate effects within specific sites of multisite trials.^21^ Distributed networks and multisite studies are unique, however, in that (1) they usually collect and combine nonrandomized data and (2) they have much more reliable access to the joint distributions of potential effect-measure modifying variables than a typical meta-analytical research project combining randomized trial results.

In this work, we explain implementation of this weighting approach when conducting pharmacoepidemiologic research in distributed networks and multisite studies and demonstrate its utility and validity in a simulated multisite study. We then apply it while imitating a past study comparing initiation of metformin or sulfonylurea as first-line treatment for type 2 diabetes within the United Kingdom’s Clinical Practice Research Datalink (CPRD) Aurum, creating an artificial distributed network consisting of a distinct site for each of the 11 regions.

## Methods

### Definitions and key concepts

#### Multisite studies and distributed networks

**Figure 1:**
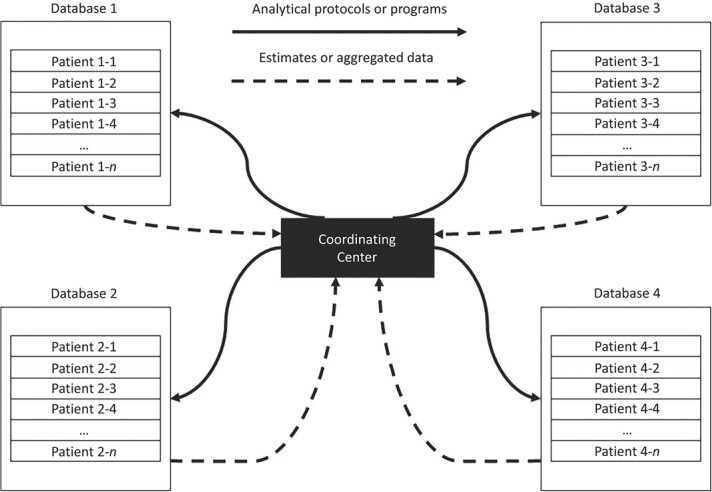
A graphical representation of the structure of multisite studies and distributed networks.

While the number of sites, the geographic diversity of the sites, data types within sites, and the amount of data returned to the coordinating center vary across networks, their structures and overall workflows are similar (Figure 1).^22^ Broadly speaking, distributed networks include a coordinating center that distributes analytical protocols or statistical code packages to sites which implement them. Site-specific results are returned to the center for further analysis, consolidation, and dissemination. Importantly, individual identifiable patient data are *not* returned to the coordinating center.

#### External validity

External validity is the extent to which the effect of an intervention $do(x)$ estimated within a study population (${P}_{\mathrm{study}}$) is not an unbiased estimate of the treatment effect in a specific target population of interest (${P}_{\mathrm{target}}$) due to differences between the ${P}_{\mathrm{study}}$ and ${P}_{\mathrm{target}}$ populations. When we have internal validity within the study population and external validity for a given target, we achieve target validity.^17^^,^^23^ External validity has similar requirements to internal validity with respect to consistency (interventions in the study population should mimic those in the target), positivity (EMMs and covariate patterns in the target population must all have a nonzero chance of being represented in the study population), and exchangeability (EMMs and covariate patterns as required by a given effect measure must be able to be balanced or otherwise accounted for).^24^ External validity is *target population-*, *outcome-*, and *effect measure-* (ie, hazard ratio vs risk difference) specific.

#### Effect measure modifiers

A binary variable *M* is an EMM for the risk difference (RD) scale effect of *X* on *Y* if it satisfies the inequality^25^


$$ P\left({Y}^{X=1}|M=1\right)-P\left({Y}^{X=0}|M=1\right)\ne P\left({Y}^{X=1}|M=0\right)-P\left({Y}^{X=0}|M=0\right) $$

and is an EMM for the risk ratio (RR) if it satisfies the inequality


$$ P\left({Y}^{X=1}|M=1\right)/P\left({Y}^{X=0}|M=1\right)\ne P\left({Y}^{X=1}|M=0\right)/P\left({Y}^{X=0}|M=0\right). $$

In simple terms, this means the effect of *X* on *Y* differs on the scale of interest across levels of *M*. While some effect measures (eg, the RD) do not require all EMMs that meet this definition to be balanced,^26^ and it can be problematic in cases of exact cancellation of modification (similar to cancellation of confounding caused by faithlessness),^25^ weighting or fitting of outcome models based on all variables meeting this definition can be a useful step toward improving external validity. Definitions for continuous *M* are similar but more complex.

#### Analytical tools for estimating an effect in a specific target

The causal structures underlying external validity issues parallel the causal structures that generate confounding and reduce internal validity.^23^^,^^27^ Just as there are many ways to standardize treated and untreated populations to one another to ensure internal exchangeability and remove confounding, there are multiple ways for researchers to standardize estimates from a given study population to a specific target. Two of the most straightforward and easy to implement are weighting (using inverse odds weights [IOWs])^14^ and outcome modeling (similar to the G formula)^28^; other methods, like doubly robust approaches, are also available.^15^ In a multisite study or distributed network, these methods can select target populations that may be of interest.

To create IOWs, we first combine the study and target populations, using an indicator variable to represent study membership. We then use regression or machine learning to estimate the probability of the indicator variable based on the variables we wish to standardize ***Z***. The IOW for each member *i* of the study population is the inverse of that member’s ***Z***-conditional odds of study participation, or


$$ {\mathrm{IOW}}_i=1/\big(\mathrm{odds}\left(\mathrm{trial}\ \right|\ \boldsymbol{Z}\big)\!\big). $$

Outcome modeling, on the other hand, is similar to using G-methods to account for confounding. First, we construct models in the study population for the probability of the outcome, ideally using separate models for each level of treatment, based on confounding variables and EMMs. After constructing these models, we predict the probability of the outcome in the target population under each level of treatment and contrast those predictions.

### Proof-of-concept simulation

#### Core simulation setup

**Figure 2:**
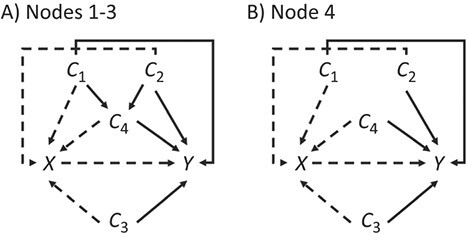
Directed acyclic graphs representing the causal structure in 4 simulated nodes included in a hypothetical distributed network. Panel A shows the first 3 sites, and panel B shows the fourth site. Solid arrows are causal effects that are present in every simulation scenario, while dashed arrows are limited to scenarios where treatment is not randomized.

We simulated a distributed network consisting of 4 sites with 10 000, 20 000, 40 000, and 80 000 individuals each to mimic real-world networks with highly variable site sizes. There were 2 independent covariates, *C*_1_ and *C*_2_, and a third independent continuous covariate, *C*_3_; there was also a fourth binary covariate, *C*_4_, associated with *C*_1_ and *C*_2_ in all but the largest population. These 4 covariates were associated with the probability of the binary outcome, *Y*. The prevalences of *C*_1_*, C*_2_, and *C*_4_ varied across the sites of the network, resulting in substantial differences in the site-specific populations. We also created a binary treatment variable, *X*. Figure 2 shows the general structure of the directed acyclic graphs at the 4 sites. Table 1 summarizes the key aspects varied within the simulation, as well as the various analytical strategies explored in the completely simulated data. Table S1 and Appendix S1 include a full list of all parameters and coefficients, as well as SAS code with which to recreate the cohorts.

**Table 1:** Dimensions explored within a proof-of-concept simulation exploring the potential utility of standardizing to specific target populations in multisite studies.

**Dimension**	**Spaces explored**
Treatment allocation	Randomized Confounded by *C*_1_, *C*_2_, *C*_3_, and *C*_4_
Scale of the outcome model	Linear Log-linear
Variables interacting with treatment	None *C*_2_ and *C*_3_
Analytical methods to adjust for confounding	IPTW
Analytical methods to standardize to a target	None (“naive”) Inverse odds weights Outcome modeling
Potential target populations	Full network/all sites Smallest site 3 smallest sites
Meta-analytical method	Inverse variance weights

Abbreviation: IPTW, inverse probability of treatment weighting.

#### Simulation parameters varied across scenarios

Because we wanted to assess a wide array of scenarios, we varied several properties of the simulation. These included (1) whether *X* was randomized or affected by *C*_1_*-C*_4_; (2) the scale of the structural equation model assigning the probability of the outcome, either linear (when studying RDs) or log-linear (when studying RRs); and (3) whether *C*_2_ and *C*_3_ modified the effect of the treatment on the scale of interest.

#### Analytical methods for internal validity

We estimated marginal effects using inverse probability of treatment weighting (IPTW), with weights estimated from a logistic regression model predicting the probability that *X* = 1 based on *C*_1_, *C*_2_, *C*_3_, and *C*_4_.

#### Target populations

We tested these methods within 3 potential target populations: (1) the full network, a common target population for distributed networks; (2) the smallest site in the network, to explore benefits of looking at specific sites; and (3) the 3 smallest sites in the network, mimicking situations where a network may have access to a large database outside the core population of interest (as is the case for CNODES and the MarketScan and CPRD Aurum databases).^6^ These 3 populations correspond to a nested study (when targeting the full network), a nonnested study (when targeting the smallest site in the network), and a partially nested study (when targeting the 3 smallest sites).^15^

#### Analytical methods for external validity

We focused on the 2 methods for standardizing to specific target populations discussed previously: IOWs (estimating probabilities of the study variable indicator based on logistic regression) and outcome modeling. We used *C*_1_*-C*_4_ in these IOWs and outcome models. Inverse probability of treatment weights and IOWs are combined by multiplying weights, and IOWs can be combined with outcome modeling via weighted generalized linear modeling. While we used a uniform approach for consistency, when conducting a nested study (ie, targeting the full network), IOWs based on probabilities from the stacking approach can perform worse than fitting inverse probability weights based on probabilities that, themselves, are estimated from weighted linear regression.^15^

#### Meta-analysis

We focused primarily on inverse variance weights (IVWs) as a meta-analysis method, since every site was estimating the same target parameter. Work in causal meta-analysis has shown that pooling via inverse variance weighting performs similarly to optimal methods that incorporate correlations in variance across sites.^29^

#### Other statistical considerations in the simulations

All variance estimates and 95% CIs came from empirical SDs across the 1000 replicates within each simulation scenario. As a “gold standard” to compare with meta-analyzed estimates, we performed analyses embedded directly within the various target populations with no regard for separating results by site.

### CPRD analyses

#### The data source and past study

The CPRD is a primary-care database of data from general practitioners covering millions of people from across the United Kingdom. Our example research question was drawn from previous studies comparing the risk of adverse outcomes including 1-year all-cause mortality after initiation of sulfonylureas and metformin as first-line treatments for type 2 diabetes.^30^^,^^31^ In addition to including more calendar time and using CPRD Aurum rather than CPRD GOLD, we did not link to the Office for National Statistics for death dates or the Hospital Episode Statistics for additional covariate information and hospital-recorded deaths so that we could include Northern Ireland, Scotland, and Wales (which do not participate in these linkages) within the study for greater diversity. We followed patients using an intention-to-treat approach with no censoring when stopping or initiating additional antidiabetic treatments after entering the cohort.^32^ This study was approved by the CPRD’s Independent Scientific Advisory Committee and the Jewish General Hospital Research Ethics Board.

#### Creating an artificial distributed network

To replicate the structure of a distributed network, we treated CPRD as if analyses had to be performed separately in 11 geographic regions. All dates and information on treatment and the outcome were kept within regions. The only information shared was the joint distribution of the potential effect measure modifying variables.

#### Target population

We focused on 1 potential target population, the smallest region (region 11, Northern Ireland). This makes our substantive example a nonnested study where IOWs and IVWs are appropriate.

#### Internal validity analyses

Within each region, we estimated treatment effects using IPTW after estimating a propensity score using a logistic regression model including a wide array of confounding variables that ranged from demographic characteristics (sex and age) to comorbid conditions (kidney disease, depression, epilepsy, left ventricular heart failure, hyperlipidemia, hypertension, arrhythmias, past cardiomyopathy, cerebrovascular disease, and others) to health behaviors (smoking and alcohol use) to concomitant medications (for chronic conditions like hypertension and atrial fibrillation and shorter-term indications like pain). See Table S2 for the full list of covariates.

#### External validity analyses

We calculated IOWs based on potential effect-measure–modifying covariates using weighted logistic regression (with the inverse probability of treatment weights acting as the weights in this regression). The specific variables we included in the IOWs were sex, age, body mass index category, coronary artery disease, duration of treated diabetes, and left ventricular heart failure. Weights were calculated separately for each treatment arm. IOWs were then multiplied by the inverse probability of treatment weights to obtain the final weights.

#### Estimating risks and combining estimates

For each region, we estimated 1-year risks of all-cause mortality for metformin and sulfonylurea initiators from weighted survival curves and estimated an RD. SEs and 95% CIs for these estimates were calculated using 2000 bootstrap replicates. These IOW-weighted RD estimates were combined using IVWs.

## Results

### Simulation results

**Figure 3:**
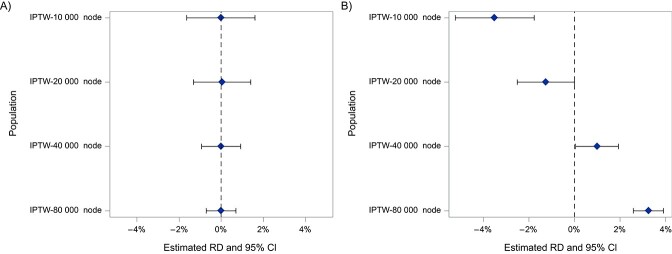
Treatment effect estimates (diamonds) across the 4 nodes in a simulation study with a confounded treatment effect. Panel A shows the estimates obtained in the absence of heterogeneity, while panel B shows estimates in the presence of heterogeneity. Bars show 95% CIs. IPTW, inverse probability of treatment weighting; RD, risk difference.

Figure 3 shows point estimates and 95% CIs for the RD within each site with a confounded treatment effect in the absence (Figure 3A) or presence (Figure 3B) of heterogeneity. Effect estimates were similar in the absence of confounding and whether outcome modeling or IPTW was used to achieve internal validity.

**Figure 4:**
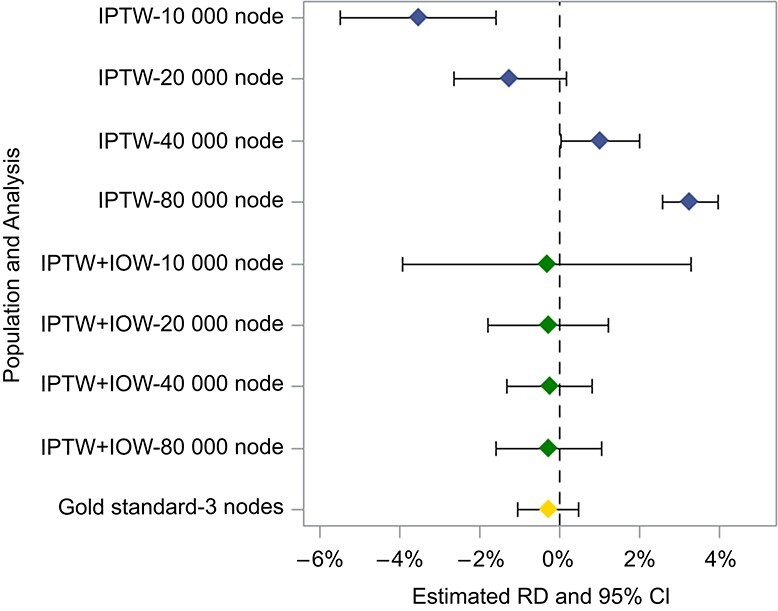
Risk differences (RDs) obtained when estimating various treatment effects using data from 4 simulated nodes and inverse probability of treatment weighting (IPTW). Blue diamonds represent the estimates within each node, green diamonds represent the estimates when standardizing each node to the covariates of the 3 smallest nodes using inverse odds weights (IOW), and yellow diamonds represent a gold standard analysis of those 3 nodes directly. Bars show 95% CIs.

**Figure 5:**
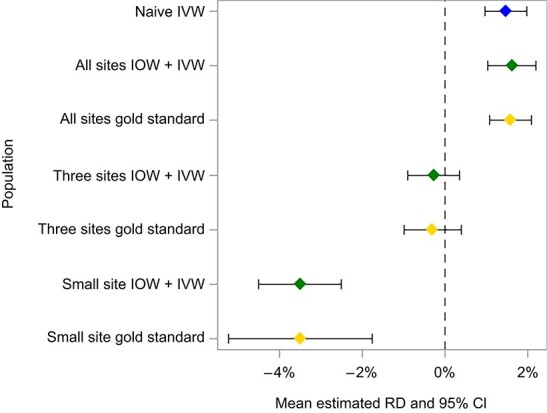
Risk differences (RDs) in various target populations among 4 simulated nodes estimated using various different methodologies. The blue diamond is the result of combining estimates with inverse variance weighting (IVW), the green diamonds are the result of combining estimates with IVW after targeting a specific population with inverse odds weights (IOW), and the gold diamonds are the result of analyzing those targets directly. Bars show 95% CIs.

Applying IOWs based on EMMs of the RD changed the magnitude and precision of treatment effect estimates. Figure 4 shows how estimates changed after applying IOWs targeting the 3 smallest sites and compares them with a “gold standard” analysis of those 3 sites. The effect estimates were all more similar after standardizing to a specific target. For all 3 targets, combining target-specific estimates using IVWs resulted in estimates centered on the “gold standard” values from analyzing the target population directly (Figure 5). The pooled estimates targeting the 3 smallest sites and the smallest site were more precise than the estimates obtained when analyzing those sites directly because they drew on data external to the target population (eg, when targeting the 3 smallest sites, we now also had some information from the largest site). Findings were similar when examining RRs.

### CPRD results

We included 813 156 metformin initiators and 193 978 sulfonylurea initiators across the 11 CPRD regions (Figure S1 shows a flow diagram for study inclusion). The probability of initiating use of sulfonylureas ranged from 18% to 21% across these regions. The smallest region (region 11) consisted of only 3146 individuals. After correcting for confounding using inverse probability of treatment weights estimated from logistic regression, the estimated 1-year intention-to-treat RD for all-cause mortality when analyzing the data combined was 2.67% (95% CI, 2.52-2.81); the treatment effect when using each region as a distinct site and combining their estimates using IVWs was 2.59% (95% CI, 2.44-2.73). This minor difference is likely attributable to the difference between fitting 1 logistic regression model and a region-stratified one.

**Figure 6:**
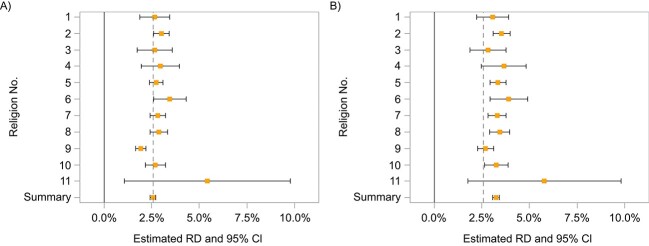
Risk difference (RD) estimates for the effect of initiating use of metformin versus sulfonylurea on 1-year mortality obtained within each region of CPRD Aurum using inverse probability of treatment weights (IPTW) (A) and when combining IPTW with inverse odds weights (IOW) to target the region 11 subpopulation (B). The dashed line represents the summary estimate obtained from applying inverse variance weights to the IPTW estimates from each region without the use of any IOWs. Bars show 95% CIs. CPRD, Clinical Practice Research Datalink.

The initial estimate of the 1-year RD in region 11 was 5.41% (95% CI, 1.03-9.79), making it the least precise and most extreme site-specific estimate. Figure 6 shows IPTW-weighted estimates from each region (Figure 6A) and how estimates changed after standardizing regions to resemble region 11 with respect to EMMs (Figure 6B). While site-specific estimates from regions 1-10 initially ranged from 1.93% to 3.46%, IOWs shifted the range from 2.70% to 3.92%. Pooling these IOW-standardized estimates together using IVWs resulted in a 1-year RD of 3.25% (95% CI, 3.07-3.43) for the smallest site—much more precise than the estimate obtained by analyzing only that site and larger than the overall effect within the network. The *I*^2^ value (the percentage of the variability in effect estimates due to heterogeneity)^33^ dropped from 71.0% to 21.4% after using IOWs. Figure S2 places these results side by side with those from a single simulation replicate bootstrapped 2000 times (*I*^2^ = 37) to illustrate what might be observed in a setting with perfect control of confounding and EMMs.

## Discussion

When using data from a variety of sources in pharmacoepidemiologic research, identifying specific target populations and standardizing each source to resemble those targets can improve the interpretability and precision of site-specific treatment effect estimates. Within our simulation, specifying target populations consistently eliminated discrepancies between the estimates across sites and, for smaller target populations, generated more precise treatment effects than analyzing those populations directly. When applying this strategy to real-world CPRD Aurum data divided into an artificial distributed network to examine a pharmacoepidemiologic research question, this approach greatly increased the precision of our treatment effect estimate at the smallest site and suggested that some of the variables used in the IOWs (sex, age, body mass index category, coronary artery disease, duration of diabetes, and left ventricular heart failure) modified the treatment effect and partially explained the elevated RD observed in the target population.

Still, these factors did not entirely explain the difference in treatment effect across the sites, since small differences in estimates still existed after standardization despite the large sample size and number of outcomes, with a final *I*^2^ value for heterogeneity of 21.4% across the sites after inverse odds weighting. While this is not entirely unexpected for a single study iteration even when our assumptions are met (as shown by Figure S2), there are likely additional unmeasured EMMs. Applying these tools with any amount of confidence will require reliable analytical methods to identify important EMMs that may differ in distribution between sites and target populations.

There is much more to be done to refine these methods, evaluate their utility, and make them better suited for pharmacoepidemiologic research questions. Many multisite studies or distributed networks have data partners that differ from one another above and beyond the specific distributions of EMMs. Some are electronic health records, some are insurance claims databases, some only include prescription information for older adults, some may have access to laboratory or genetic markers, and so on. Our substantive example was not intended to be a substitute for exploring these methods in a real distributed network. Unlike a real distributed network, it did not have large differences in treatment distribution or cultural differences between sites, and it had greater homogeneity in general than typical distributed networks. Testing the feasibility of these methods in studies in real distributed networks is a key part of validating their performance. One place where these concerns about data source heterogeneity may be limited is applications of these methods to improve the precision of treatment effect estimates within subgroups when researchers can identify the EMMs that differ between that subgroup and the rest of the study population.

This approach is not without significant limitations and assumptions, however. If important EMMs are ignored, estimates for some target populations will be biased. Just as one cannot verify assumptions of no unmeasured confounding in nonexperimental research, one cannot verify whether they have unmeasured EMMs. In our example, suppose that specific sulfonylureas were especially unsafe, and their use differed across regions; we would need to incorporate drug type into our IOWs. Moreover, if the variable separating data into unique analytical units (like region in our example) modifies the treatment effect conditional on measured variables, standardization will not work. If there is only 1 region where individuals receive a drug therapy that has a negative interaction with sulfonylureas, for example, no amount of inverse odds weighting will be able to achieve external validity for that region. If researchers are unsure whether necessary assumptions are valid, however, they can use sensitivity analyses incorporating that uncertainty^34^ or bayesian approaches to combining estimates.^35^

These methods also rely on achieving internal validity within the sites that make up the distributed network or full study population. Measurement error can differ between sites. In our example, some regions in CPRD Aurum may be less likely to capture deaths in the primary-care records than others, with worse sensitivity for death resulting in smaller observed RDs. Similarly, missing data may only affect 1 site because of differing rules on collecting laboratory values before starting treatment (eg, if Northern Ireland required collecting 2 hemoglobin A1C measures before starting treatment for diabetes, while other regions did not). Selection bias and loss to follow-up can also differ dramatically from one data source to another: In our example, some regions could have more mobility in and out of the CPRD general practitioner practices. Finally, residual and unmeasured confounding can obviously differ across sites.

This all makes it very difficult to distinguish between unmeasured biasing factors for internal and external validity. Sensitivity analyses, validation studies, and data-source–specific investigations such as evaluating the sensitivity and specificity for death using linkages of CPRD Aurum to the Office for National Statistics will be key to using these methods to make specific policy and regulatory decisions. Additionally, the different coding systems and types of data captured by each data source (eg, CPRD capturing prescriptions ordered by general practitioners, while insurance claims capture prescriptions actually filled by patients) can mean that sites are estimating subtly different treatment effects that in reality may not be appropriate to combine with one another. Researchers must always be cautious about combining estimates that wildly differ from one another, especially after measured EMMs have been taken into account.

The greatest practical limitation of this approach is that it only works if sites share information on the distribution of EMMs (or analytical models) with the coordinating center and with other sites. Even if data sets are anonymized with no information on dates, times, treatment arms, or outcome rates, some may resist sharing joint distributions. That said, sites reluctant to share data can still participate in analyses without receiving the benefits enjoyed by the other sites. When privacy is a concern, generative adversarial networks^36^ can create synthetic data sets similar to the original site populations without sharing proprietary or identifiable information. Moreover, under additional assumptions, sharing marginal rather than joint distributions of EMMs (the type of information commonly published in research articles) may be sufficient.^37^

## Conclusion

While the method is not without major limitations, identifying and standardizing data to specific target populations has the potential to increase the precision and interpretability of pharmacoepidemiologic research using multiple sites or distributed networks. Even if some assumptions are not met, these analyses can aid in understanding why estimates differ between segments of a study population. That said, routinely using this approach in distributed networks requires additional methodological and substantive work in real networks to evaluate the extent to which assumptions are violated and robustness to violations of those assumptions.

## Supplementary Material

Web_Material_kwae015
