# Supplementary material for: Standardizing to specific target populations in distributed networks and multisite pharmacoepidemiologic studies
Source: Am J Epidemiol. 2024 Feb 27;193(7):1031–9. doi: 10.1093/aje/kwae015 (PMC11520739; doi:10.1093/aje/kwae015)
Supplement: Web_Material_kwae015 [file web_material_kwae015.zip › kwae015 Platt Appendix S1.docx]

**APPENDIX S1**

**Cohort Creation SAS Code**

(Additional cohort creation and analytical code is available upon request.)

1. Code for creating a single node:

**%macro** nodemake( nodename=,seed=,size=,

C1prev=,C2prev=,C3mean=,C3stddev=,

truncC3=, truncC3val=,

intC4=,C2ORC4=,C3ORC4=,

intexp=,C1ORexp=,C2ORexp=,C3ORexp=,C4ORexp=,

outscale=,

intout=,expout=,C1cofout=,C2cofout=,C3cofout=,C4cofout=,C2expcofout=,C3expcofout=);

DATA &nodename;

CALL streaminit(&seed);

LENGTH network $ **40**;

network="&nodename";

DO ID = **1** to &size;

C1=RAND("BERNOULLI",&C1prev);

C2=RAND("BERNOULLI",&C2prev);

C3=RAND("NORMAL",&C3mean,&C3stddev);

logitC4=log(&intC4) + C2*log(&C2ORC4) + C3*log(&C3ORC4);

oddsC4=exp(logitC4);

probC4=oddsC4/(**1**+oddsC4);

C4=RAND("BERNOULLI",probC4);

logitexp=log(&intexp) + C1*log(&C1ORexp) + C2*log(&C2ORexp) + C3*log(&C3ORexp);

oddsexp=exp(logitexp);

probexp=oddsexp/(**1**+oddsexp);

exp=RAND("BERNOULLI",probexp);

%IF &outscale = identity %THEN %DO;

probout= &intout + exp*&expout + C1*&C1cofout + C2*&C2cofout + C3*&C3cofout + C4*&C4cofout + C2*exp*&C2expcofout + C3*exp*&C3expcofout;

%END;

%ELSE %DO;

logprobout= log(&intout) + exp*log(&expout) + C1*log(&C1cofout) + C2*log(&C2cofout) + C3*log(&C3cofout) + C4*log(&C4cofout) + C2*exp*log(&C2expcofout) + C3*exp*log(&C3expcofout);

probout = exp(logprobout);

%END;

IF probout > **1** THEN probouttrunc = **1**;

ELSE IF probout < **0** THEN probouttrunc = **0**;

ELSE probouttrunc = probout;

out=RAND("BERNOULLI",probouttrunc);

OUTPUT;

%IF &truncC3 = yes %THEN %DO;

IF C3 <= &truncC3val THEN DELETE;

%END;

END;

KEEP exp C1 C2 C3 C4 out network probout;

RUN;

**%mend**;

2. Code for creating multiple nodes:

**%macro** multinodes(startrep=,endrep=,startseed=,

overallname=,

truncC3=,truncC3val=,

intexp=,C1ORexp=,C2ORexp=,C3ORexp=,C4ORexp=,

scale=,

intout=,expout=,C1cofout=,C2cofout=,C3cofout=,C4cofout=,

C2expcofout=,C3expcofout=);

%DO rep = &startrep %TO &endrep ;

%***nodemake***(nodename=&overallname.1,seed=&startseed + **1**+ &rep.***4**,size=**10000**,

C1prev=**0.2**,C2prev=**0.2**,C3mean=**0**,C3stddev=**1**,

truncC3=no,truncC3val=**.**,

intC4=**1.0**,C2OrC4=**1.5**,C3ORC4=**1.1**,

intexp=&intexp,C1ORexp=&C1ORexp,C2ORexp=&C2ORexp,C3ORexp=&C3ORexp,C4ORexp=&C4ORexp,

outscale=&scale,

intout=&intout,expout=&expout,C1cofout=&C1cofout,C2cofout=&C2cofout,C3cofout=&C3cofout,C4cofout=&C4cofout,

C2expcofout=&C2expcofout,C3expcofout=&C3expcofout);

%***nodemake***(nodename=&overallname.2,seed=&startseed + **2** + &rep.***4**,size=**20000**,

C1prev=**0.4**,C2prev=**0.4**,C3mean=**0.25**,C3stddev=**1**,

truncC3=no,truncC3val=**.**,

intC4=**0.5**,C2OrC4=**1.5**,C3ORC4=**1.1**,

intexp=&intexp,C1ORexp=&C1ORexp,C2ORexp=&C2ORexp,C3ORexp=&C3ORexp,C4ORexp=&C4ORexp,

outscale=&scale,

intout=&intout,expout=&expout,C1cofout=&C1cofout,C2cofout=&C2cofout,C3cofout=&C3cofout,C4cofout=&C4cofout,

C2expcofout=&C2expcofout,C3expcofout=&C3expcofout);

%***nodemake***(nodename=&overallname.3,seed=&startseed + **3** + &rep.***4**,size=**40000**,

C1prev=**0.6**,C2prev=**0.6**,C3mean=**0.5**,C3stddev=**1**,

truncC3=no,truncC3val=**.**,

intC4=**0.7**,C2OrC4=**1.5**,C3ORC4=**1.1**,

intexp=&intexp,C1ORexp=&C1ORexp,C2ORexp=&C2ORexp,C3ORexp=&C3ORexp,C4ORexp=&C4ORexp,

outscale=&scale,

intout=&intout,expout=&expout,C1cofout=&C1cofout,C2cofout=&C2cofout,C3cofout=&C3cofout,C4cofout=&C4cofout,

C2expcofout=&C2expcofout,C3expcofout=&C3expcofout);

%***nodemake***(nodename=&overallname.4,seed=&startseed + **1**+ &rep.***4**,size=**80000**,

C1prev=**0.8**,C2prev=**0.8**,C3mean=**0.75**,C3stddev=**1**,

truncC3=no,truncC3val=**.**,

intC4=**1**,C2OrC4=**1.0**,C3ORC4=**1.0**,

intexp=&intexp,C1ORexp=&C1ORexp,C2ORexp=&C2ORexp,C3ORexp=&C3ORexp,C4ORexp=&C4ORexp,

outscale=&scale,

intout=&intout,expout=&expout,C1cofout=&C1cofout,C2cofout=&C2cofout,C3cofout=&C3cofout,C4cofout=&C4cofout,

C2expcofout=&C2expcofout,C3expcofout=&C3expcofout);

**%mend**;

3. Code for creating 4 nodes with no confounding and a linear outcome model with no EMM:

%multinodes( startrep=201,endrep=400,startseed=1,

overallname=randlinnointx,

intexp=1,C1ORexp=1,C2ORexp=1,C3ORexp=1,C4ORexp=1,

scale=identity,

intout=0.22,expout=0,C1cofout=0.1,C2cofout=0.1,C3cofout=0.03,C4cofout=0.1,

C2expcofout=0.0,C3expcofout=0.0);

4. Code for creating 4 nodes with no confounding, a linear outcome model, and EMM:

%multinodes( startrep=801,endrep=1000,startseed=10000,

overallname=randlinintx,

intexp=1,C1ORexp=1,C2ORexp=1,C3ORexp=1,C4ORexp=1,

scale=identity,

intout=0.22,expout=-0.05,C1cofout=0.1,C2cofout=0.1,C3cofout=0.03,C4cofout=0.1,

C2expcofout=0.075,C3expcofout=0.03);

5. Code for creating 4 nodes with confounding and a log-linear outcome model with EMM:

%***multinodes***( startrep=**401**,endrep=**1000**,startseed=**9110000**,

overallname=conflogintx,

intexp=**0.3**,C1ORexp=**1.5**,C2ORexp=**1.5**,C3ORexp=**1.1**,C4ORexp=**1.5**,

scale=log,

intout=**0.30**,expout=**0.75**,C1cofout=**0.6**,C2cofout=**0.6**,C3cofout=**0.90**,C4cofout=**0.8**,

C2expcofout=**2.0**,C3expcofout=**1.05**);
