# Supplementary material for: Standardizing to specific target populations in distributed networks and multisite pharmacoepidemiologic studies
Source: Am J Epidemiol. 2024 Feb 27;193(7):1031–9. doi: 10.1093/aje/kwae015 (PMC11520739; doi:10.1093/aje/kwae015)
Supplement: Web_Material_kwae015 [file web_material_kwae015.zip › kwae015 Platt Supplementary Material.pdf]

SUPPLEMENTARY MATERIAL

Standardizing to Specific Target Populations in Distributed Networks and Multisite Pharmacoepidemiologic Studies

Michael Webster-Clark, Kristian B. Filion, and Robert W. Platt

Table of Contents

Table S1: Simulation parameters ________________________________ Page 2

Appendix S1: Cohort creation SAS code________________________ (Word file)

Table S2: Measured confounding variables________________________ Page 3

Figure S1: Cohort flow diagram________________________________ Page 4

Figure S2: Substantive vs simulation results________________________ Page 5

Table S1: Simulation parameters

Parameter	Site 1 Value	Site 2 Value	Site 3 Value	Site 4 Value
Population size	10,000	20,000	40,000	80,000
Prevalence of C_1	20%	40%	60%	80%
Prevalence of C_2	20%	40%	60%	80%
C_3 mean	0	0.25	0.50	0.75
C_3 standard deviation	1	1	1	1
C_4 base odds	1	0.5	0.7	1
C_2 OR for C_4	1.5	1.5	1.5	1.0
C_3 OR for C_4	1.1	1.1	1.1	1.0
Exposure model (if creating confounding)	$\text{Log}(\text{odds}(X))=\log(0.3) + \log(1.5)*C_1 + \log(1.5)*C_2 + \log(1.1)*C_3 + \log(1.5)*C_4$			
Outcome model (linear, no EMM)	$\text{Probability}(Y)=0.22 + 0.1*C_1 + 0.1*C_2 + 0.03*C_3 + 0.1*C_4$			
Outcome model (linear, EMM)	$\text{Probability}(Y)=0.22 + 1*C_1 + 0.1*C_2 + 0.03*C_3 + 0.1*C_4 - 0.05*X + 0.075*C_2*X + 0.03*C_3*X$			
Outcome model (log, no EMM)	$\text{Log}(\text{Probability}(Y))=\log(0.30) + \log(0.6)*C_1 + \log(0.6)*C_2 + \log(0.90)*C_3 + \log(0.8)*C_4$			
Outcome model (log, EMM)	$\text{Log}(\text{Probability}(Y))=\log(0.30) + \log(0.6)*C_1 + \log(0.6)*C_2 + \log(0.90)*C_3 + \log(0.8)*C_4 +\log(0.70)*X + \log(2.0)*X*C_2 + \log(1.05)*X*C_3$			

OR=odds ratio; EMM=effect measure modifier.

Table S2: List of measured confounding variables in the empirical example.

Type of Covariate	Specific Covariates Included
Demographics	Gender (binary)<br>Calendar year (continuous)<br>Age (continuous, with square term)
Health status	Body mass index (categorical)<br>Smoking (categorical)<br>Alcohol use (binary)<br>Hemoglobin A1C (categorical)<br>Diabetes duration
Specific comorbidities	Arrythmias (binary)<br>Cardiomyopathy (binary)<br>Cerebrovascular (binary)<br>Kidney disease (binary)<br>Coronary artery disease (binary)<br>Depression (binary)<br>Epilepsy (binary)<br>Conductivity disorders (binary)<br>Heart failure (binary)<br>Hyperlipidemia (binary)<br>Hypertension (binary)<br>Hypomagnesemia (binary)<br>Hypocalcemia (binary)<br>Hypokalemia (binary)<br>Left ventricular heart failure (binary)<br>Liver disease (binary)<br>Peripheral vascular disease (binary)<br>Valve replacement (binary)
Co-medications	Acetaminophen use (binary)<br>ACE inhibitor (binary)<br>Angiotension receptor blockers (binary)<br>Anitarrythmic drugs (binary)<br>Aspirin (binary)<br>Beta blockers (binary)<br>Calcium channel blockers (binary)<br>Clopidogrel (binary)<br>Digoxin (binary)<br>Direct oral anticoagulants (binary)<br>Fibrates (binary)<br>Integrase inhibitors (binary)<br>NSAIDs (binary)<br>Thiazides (binary)<br>Opioids (binary)<br>Statins (binary)<br>Warfarin use (binary)<br>Any antihypertensive (binary)

Figure S1: Cohort flow diagram

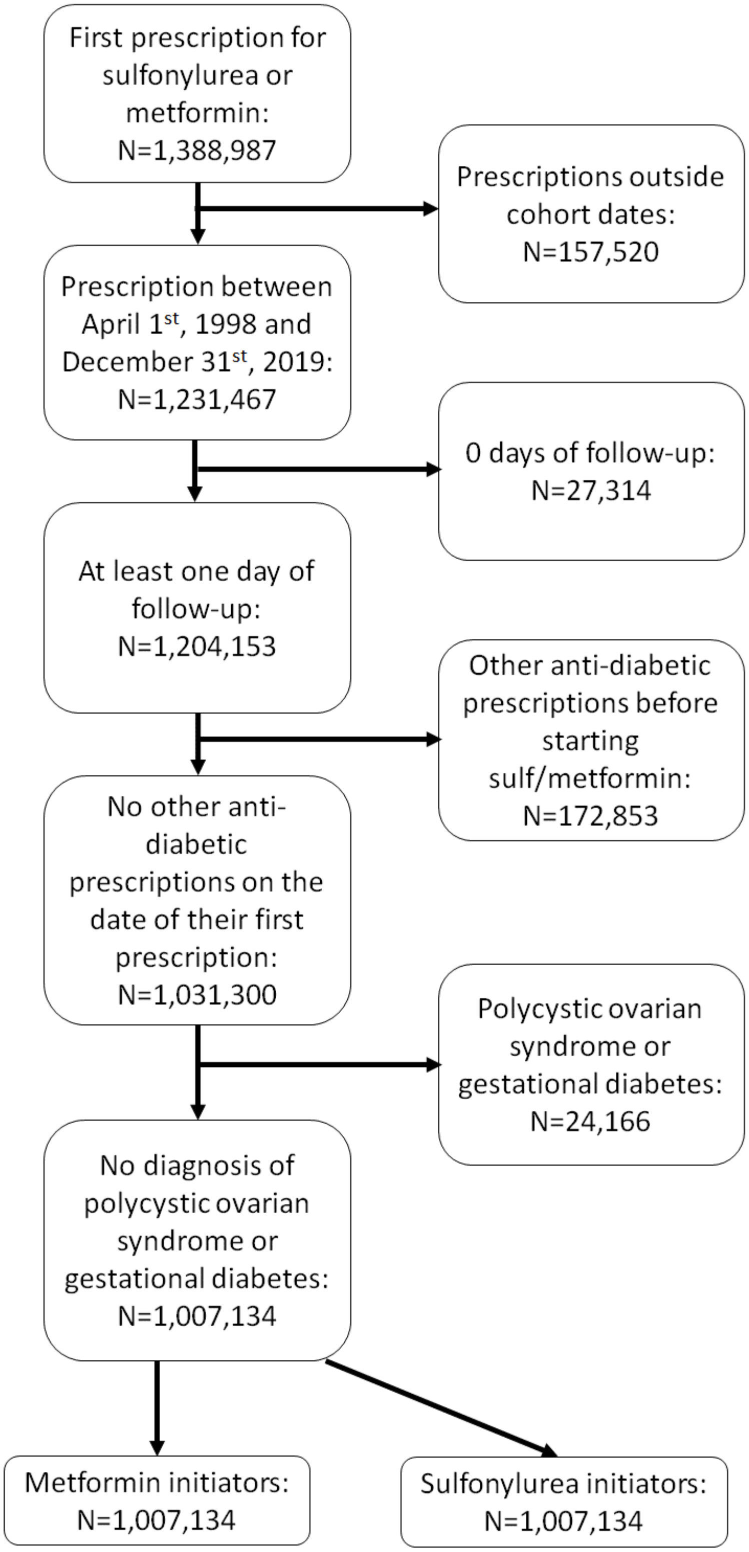

Figure S2: Comparing results from a single iteration of the simulation study with no unmeasured confounding or EMM bootstrapped 2000 times (A) to the IOW-weighted results from the substantive example (B).

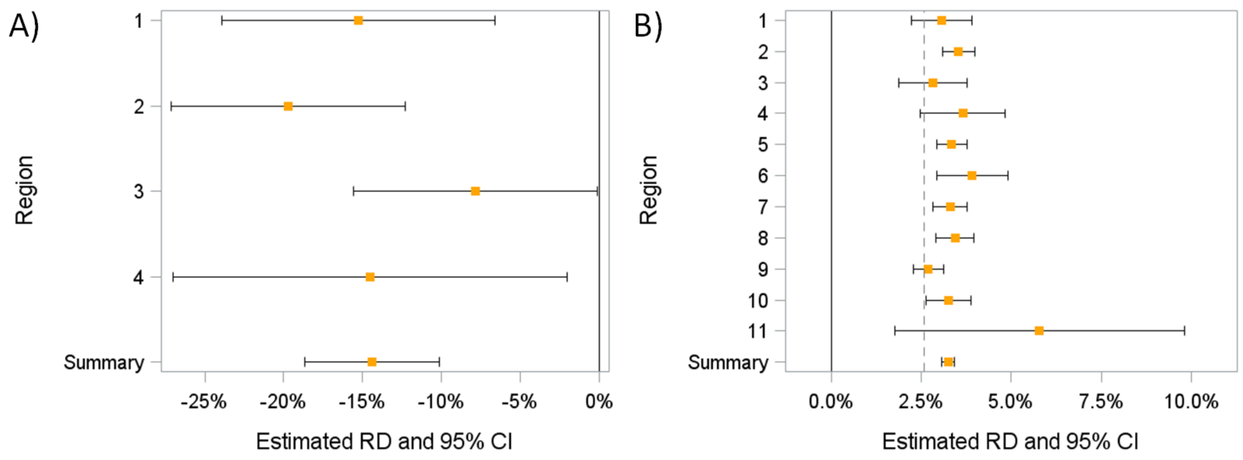

RD, risk difference. Bars, 95% CI.
